# RETRACTION: Long Non‐Coding RNA TGLC15 Advances Hepatocellular Carcinoma by Stabilizing Sox4

**DOI:** 10.1002/jcla.70002

**Published:** 2025-02-05

## Abstract

**RETRACTION**: Y. Chen, F. Huang, L. Deng, Y. Tang, D. Li, T. Wang, Y. Fan, Q. Tao, and D. Tang, “Long Non‐Coding RNA TGLC15 Advances Hepatocellular Carcinoma by Stabilizing Sox4,” *Journal of Clinical Laboratory Analysis* 34, no. 1 (2020): e23009, https://doi.org/10.1002/jcla.23009.

The above article, published online on 08 September 2019 in Wiley Online Library (wileyonlinelibrary.com), has been retracted by agreement between the journal Editor‐in‐Chief, Rong Fu; and Wiley Periodicals LLC. The retraction has been agreed upon following an investigation into concerns raised by a third party, which revealed inappropriate duplication of image panels (Figure 2E) between this article and several other articles that were previously published in a different scientific context. Thus, the editors consider the conclusions of this manuscript substantially compromised.

D. Tang, listed as the corresponding author, asked to retract the article and stated that they were not involved in or given consent to this publication and the corresponding email address mentioned under their name does not belong to them.

**RETRACTION**: Y. Chen, F. Huang, L. Deng, Y. Tang, D. Li, T. Wang, Y. Fan, Q. Tao, and D. Tang, “Long Non‐Coding RNA TGLC15 Advances Hepatocellular Carcinoma by Stabilizing Sox4,” *Journal of Clinical Laboratory Analysis* 34, no. 1 (2020): e23009, https://doi.org/10.1002/jcla.23009.

The above article, published online on 08 September 2019 in Wiley Online Library (wileyonlinelibrary.com), has been retracted by agreement between the journal Editor‐in‐Chief, Rong Fu; and Wiley Periodicals LLC. The retraction has been agreed upon following an investigation into concerns raised by a third party, which revealed inappropriate duplication of image panels (Figure 2E) between this article and several other articles that were previously published in a different scientific context. Thus, the editors consider the conclusions of this manuscript substantially compromised.

D. Tang, listed as the corresponding author, asked to retract the article and stated that they were not involved in or given consent to this publication and the corresponding email address mentioned under their name does not belong to them.

